# Draft Genome Sequence of a Fish Pathogen, *Edwardsiella piscicida* Isolate CK41

**DOI:** 10.1128/MRA.00061-20

**Published:** 2020-04-23

**Authors:** Se-Won Baek, Sungmin Hwang, Ho Young Kang, Woo Young Bang, Ki Hwan Moon

**Affiliations:** a Division of Marine Bioscience, Korea Maritime & Ocean University, Busan, Republic of Korea; b Department of Biology, Duke University, Durham, North Carolina, USA; c Department of Microbiology, Pusan National University, Busan, Republic of Korea; d National Institute of Biological Resources, Environmental Research Complex, Incheon, Republic of Korea; University of Arizona

## Abstract

Edwardsiella piscicida CK41 is a fish-pathogenic Gram-negative bacterium isolated from diseased flounder in the Republic of Korea. Here, we report the genome sequence of *E. piscicida* CK41, comprising one chromosome of 3.76 Mbp and one plasmid of 72.7 kbp. A total of 3,406 protein-coding genes, 98 tRNAs, and 25 rRNAs are predicted to be present in the genome.

## ANNOUNCEMENT

The Gram-negative bacterial genus *Edwardsiella* consists of five species; Edwardsiella hoshinae, E. ictaluri, E. anguillarum, E. tarda, and E. piscicida are known to cause fish diseases (1). *E. piscicida* is one of the well-studied species in the family, as it is a notorious aquatic pathogen and causes economic damage to the fisheries industry. The *Edwardsiella* isolate CK41, which was used in this study, was originally isolated from a diseased flounder (2). *E. piscicida* was initially classified as an *E. tarda*-like species but later was described as a new distinct species based on phylogenetic analysis by Abayneh et al. in 2013 (3). Since then, some E. tarda strains were reclassified as E. piscicida, including E. piscicida strain CK41, which was sequenced in this study.

A single colony from the *E. piscicida* CK41 isolate was grown until the exponential phase and harvested for the genomic DNA using a HiGene genomic DNA prep kit (Biofact, Daejeon, South Korea). A total of 5 μg genomic DNA was sheared, and the small fragments of less than 20 kb were removed using the Blue Pippin size selection system (Sage Science, Beverly, MA, USA). The sequencing library was constructed using a SMRTbell template prep kit version 1.0 (Pacific Biosciences, Menlo Park, CA, USA) following the manufacturer’s instructions. After primer annealing and polymerization using a DNA/polymerase binding kit P6 (Pacific Biosciences, Menlo Park, CA, USA), the genome sequence (total of 273,475 reads and 1,585,762,413 bp) of *E. piscicida* CK41 was obtained through a PacBio RS II single-molecule real-time (SMRT) cell platform (Pacific Biosciences, Menlo Park, CA, USA). The processing of the raw PacBio reads and *de novo* assembly were conducted using the Hierarchical Genome Assembly Process (HGAP) version 2.3 with default parameters, followed by multiple rounds of polishing with Quiver until a highly accurate consensus for the final assembly was derived (4). The draft genome sequence of *E. piscicida* CK41 comprised two contigs with an *N*_50_ value of 3,780,188 bp and a coverage depth of 225×. In addition, the comparisons between the previously sequenced *E. piscicida* strains with the CK41 contigs in this study were performed using MUMmer version 3.5 (5). The length and GC content of contig 1 were 3,761,749 bp and 59.73%, respectively, while those of contig 2 were 72,748 bp and 53.91%, respectively. The draft genome sequence of *E. piscicida* CK41 was annotated using the NCBI Prokaryotic Genome Annotation Pipeline (6), yielding 3,406 protein-coding sequences, 98 tRNAs, and 25 rRNAs. Average nucleotide identity (ANI) analysis was performed with the ANI calculator (http://enve-omics.ce.gatech.edu/ani/), and 99.41% of the DNA sequence was identical to a representative *E. piscicida* strain, C07-087 (7).

### Data availability.

The draft genome sequence of *Edwardsiella piscicida* CK41 has been deposited in GenBank under the accession numbers CP047671 and CP047672, for its chromosome and plasmid, respectively. The associated BioProject and BioSample accession numbers are PRJNA600308 and SAMN13812492, respectively. The raw sequence reads were deposited in the SRA under accession number SRR10906467.
